# Global Genome Response of *Escherichia coli* O157∶H7 Sakai during Dynamic Changes in Growth Kinetics Induced by an Abrupt Temperature Downshift

**DOI:** 10.1371/journal.pone.0099627

**Published:** 2014-06-13

**Authors:** Thea King, Chawalit Kocharunchitt, Kari Gobius, John P. Bowman, Tom Ross

**Affiliations:** 1 Commonwealth Scientific and Industrial Research Organisation, Animal, Food and Health Sciences, North Ryde, New South Wales, Australia; 2 Food Safety Centre, Tasmanian Institute of Agriculture, University of Tasmania, Hobart, Tasmania, Australia; 3 Commonwealth Scientific and Industrial Research Organisation, Animal, Food and Health Sciences, Werribee, Victoria, Australia; Indian Institute of Science, India

## Abstract

*Escherichia coli* O157∶H7 is a mesophilic food-borne pathogen. We investigated the growth kinetics of *E. coli* O157∶H7 Sakai during an abrupt temperature downshift from 35°C to either 20°C, 17°C, 14°C or 10°C; as well as the molecular mechanisms enabling growth after cold stress upon an abrupt downshift from 35°C to 14°C in an integrated transcriptomic and proteomic analysis. All downshifts caused a lag period of growth before growth resumed at a rate typical of the post-shift temperature. Lag and generation time increased with the magnitude of the shift or with the final temperature, while relative lag time displayed little variation across the test range. Analysis of time-dependent molecular changes revealed, in keeping with a decreased growth rate at lower temperature, repression of genes and proteins involved in DNA replication, protein synthesis and carbohydrate catabolism. Consistent with cold-induced remodelling of the bacterial cell envelope, alterations occurred in the expression of genes and proteins involved in transport and binding. The RpoS regulon exhibited sustained induction confirming its importance in adaptation and growth at 14°C. The RpoE regulon was transiently induced, indicating a potential role for this extracytoplasmic stress response system in the early phase of low temperature adaptation during lag phase. Interestingly, genes previously reported to be amongst the most highly up-regulated under oxidative stress were consistently down-regulated. This comprehensive analysis provides insight into the molecular mechanisms operating during adaptation of *E. coli* to growth at low temperature and is relevant to its physiological state during chilling in foods, such as carcasses.

## Introduction

The enterohemorrhagic *Escherichia coli* (EHEC) pathotype is comprised of a diverse group of Shiga toxin-producing *E. coli* (STEC) strains that cause severe disease, including the potentially fatal haemolytic uremic syndrome (HUS). *E. coli* O157∶H7 is one of the most important EHEC serotypes in relation to public health, contributing significantly to human infections and outbreaks. The major transmission route for O157∶H7 is food-borne, with many different foods and dairy products acting as vectors in outbreaks and sporadic cases of infection [1]. However, the majority of reported cases appear to be attributed to the consumption of foods of bovine origin, with ground beef the most frequently identified vehicle [1]. Cattle are the primary reservoir of O157∶H7 and this organism can be found in their faeces, rumen, hide, derived carcasses and meat products [2], [3], [4]. Persistence of this pathogen in the agricultural and food processing industry environments poses potential risks for human health.

Refrigeration is the most common means of preserving food in developed nations, either alone or in combination with other methods such as the addition of preservatives [5]. The ability to survive or grow at low temperature is therefore inherently important to many food-borne mesophilic bacterial pathogens, including *E. coli*. *E. coli* is able to grow at temperatures as low as 8°C, although the optimum growth temperature is around 37°C [6]. During transition to low temperature, bacteria must overcome a number of chemico-physical changes, including decreased membrane fluidity, which reduces nutrient uptake capabilities; increased superhelical coiling of DNA, which can negatively affect a bacterium's ability to replicate or transcribe DNA; stabilization of the secondary structures of RNA, which affect translation; reduced enzyme activities; inefficient or slow protein folding; and reduced ribosome function (for review, see [7]). The response of exponentially growing bacterial cells to non-lethal cold shock conditions above the minimal growth temperature has been divided into three stages; the first stage represents the initial but transient cold shock response that immediately follows cold exposure and may last several hours during which a profound reduction in the growth rate as well as marked reprogramming of protein synthesis occurs, the second stage involves a restoration of a balanced exponential growth rate and further modification of protein synthesis where cells are cold-adapted and, the third stage is where cells reach stationary phase and gene expression is altered again [8]. Most molecular studies to date have focused on the response of bacterial cells to the transient cold shock response [9], [10]. However, a comprehensive understanding of the molecular response of *E. coli* during adaptation and growth at low temperature is vital if we are to design targeted processing and preservation strategies to effectively control this pathogen.

In a previous study we adopted an integrated transcriptomic and proteomic approach to determine the physiological response of *E. coli* O157∶H7 Sakai during exponential phase growth under steady-state conditions relevant to low temperature and water activity conditions experienced during meat carcass chilling in cold air [11]. The findings of that study provide a baseline of knowledge of the physiology of this pathogen, with the response of *E. coli* O157∶H7 to steady-state conditions of combined cold and osmotic stress (14°C a_w_ 0.967 and 14°C a_w_ 0.985), such as occurs during carcass chilling. Activation of the master stress response regulator RpoS and the Rcs phosphorelay system involved in the biosynthesis of the exopolysacharide colanic acid, as well as down-regulation of genes and proteins involved in chemotaxis and motility were observed [11]. To provide an insight into the genetic systems enabling this organism to adapt to growth at low temperature, we extended the aforementioned study to investigate the growth kinetics of *E. coli* O157∶H7 Sakai during abrupt temperature downshift from 35°C to either 20°C, 17°C, 14°C or 10°C and, examined time-dependent global alterations in its genome expression upon cold shock from 35°C to 14°C. The genome-wide expression response of *E. coli* was analysed by both cDNA microarray (transcriptome response) and 2D-LC/MS/MS analysis (proteome response). Differences in gene and protein expression patterns in *E. coli* before and after cold shock were analysed through quantitative and comparative analysis of time series changes in both mRNA and proteins levels.

## Materials and Methods

### Division of experimental work

Growth rate experiments studying the response of *E. coli* O157∶H7 to temperature downshift were conducted in the Food Safety Centre (Hobart, Australia). Parallel transcriptomic and proteomic studies were conducted in independent laboratories; transcriptome studies were conducted at CSIRO Animal, Food and Health Sciences (North Ryde, Australia) and proteome studies at the Food Safety Centre, University of Tasmania (Hobart, Australia), respectively. The differences in the protocol followed at each site pertain to the different methods employed for RNA and protein extraction and identification, as well as the use of different equipment for optical density measurements (Transcriptomic: Pharmacia Biotech Novaspec II spectrophotometer; and Proteomic: Spectronic 20, Bausch and Lomb) and waterbaths for incubation of cultures (Transcriptomic: Julabo SW23 waterbath (80 rpm); and Proteomic: Ratek SWB20D shaking waterbath (80 oscillations.min^−1^)).

### Bacterial strain and preparation of inocula

To mimic experiments conducted previously [11], *E. coli* O157∶H7 strain Sakai [12], [13] obtained from Dr. Carlton Gyles (Guelph, Canada) was used throughout this study and stored at −80°C. A stock culture was cultivated on brain-heart infusion (BHI) agar at 37°C for 24 h. A well-isolated single colony from a BHI agar plate was used to inoculate 25 ml of fresh BHI broth, and incubated at 37°C without shaking for 20 h. This culture was stored at 4°C and used as a ‘working’ culture within a week.

### Growth responses after downshifts in temperature

The working culture was diluted 1∶10^4^ in 25 ml of pre-warmed (35°C) BHI broth to prepare a ‘primary’ culture. This culture was incubated at 35°C with agitation (80 oscillations.min^−1^; Ratek Instruments, Boronia, Australia) and growth was monitored turbidimetrically at 600 nm with a Spectronic 20 spectrophotometer (Bausch and Lomb, Rochester, USA). After achieving an OD_600_ of 0.1±0.01 (i.e. the exponential phase of growth, ∼10^7^ CFU.ml^−1^), the culture was diluted 1∶100 in 25 ml of sterile pre-warmed (35°C) BHI broth to prepare a ‘secondary’ culture. This culture was re-incubated at 35°C with agitation to the exponential growth phase (OD_600_ of 0.1±0.01). At this point the culture was abruptly shifted to a refrigerated water bath held either at 20°C, 17°C, 14°C or 10°C. Water baths were maintained within ±0.1°C of the test temperature.

### Microbiological analysis

Growth of *E. coli* was determined by measuring percent transmittance (%T) at 600 nm. The %T values were converted to optical density (OD) values. All experiments were conducted at least twice and the results are reported as the average of the replicates.

Growth curves were constructed by plotting log_10_ OD versus time. Linear regression analysis of the growth curve data was then used to estimate the generation time (GT) and lag time (LT), according to methods of Mellefont et al. [14]. Briefly, a straight line was fitted to those data points that best represent the exponential phase of growth. To ensure the accuracy of this estimation, at least four time points over this growth phase were used and the R^2^ value for the regression lines was ≥0.95. The GT was then calculated by dividing 0.301 (equivalent to log_10_ 2) by the slope of the line. The LT was estimated from the time at which the regression line through the exponential part of the growth curve first exceeded the initial log_10_ OD after applying the temperature downshift (i.e. the time taken to increase above starting numbers). To assess the amount of “work” required by *E. coli* to adjust to a new temperature, relative lag time (RLT) was calculated by dividing LT by GT [15], [16].

### Preparation of samples for transcriptomic and proteomic analysis

In separate cold shock experiments, cultures were subjected to a rapid temperature downshift from 35°C to 14°C as described above to prepare samples for cDNA microarray and 2D-LC/MS/MS analysis. An appropriate volume of each of the cell cultures was harvested before cold shock (i.e. the reference culture), and at 30, 90, 160, and 330 min post-temperature downshift. Independent samples were then subjected to extraction of RNA, and both soluble and membrane proteins, according to the methods of Kocharunchitt et al. [11]. The number of biological replicates taken at each time point for the transcriptomic and proteomic analysis is described in Table 1.

**Table 1:** The number of valid protein identifications and significantly differentially expressed genes and proteins after temperature downshift of *E. coli* O157∶H7 Sakai from 35°C to 14°C.

Time points (min)	Number of biological replicates		Number of proteins^b^			Number of differentially expressed genes and proteins^d^		
	Transcriptome	Proteome^a^	Membrane fraction	Soluble fraction	Total fraction	Trancriptome (total)^c^	Proteome (total)	Transcriptome *vs.* Proteome^e^
**Before downshift**	3	6(6)	343	1,320	1,370	NA^e^	NA	NA
**30 min after the shift**	3	2(2)	704	824	1,074	503(292)	137(20)	13(3)
**90 min after the shift**	3	2(2)	717	864	1,107	637(466)	155(26)	26(2)
**160 min after the shift**	3	2(2)	702	890	1,115	594(378)	118(25)	22(6)
**330 min after the shift**	3	2(2)	858	864	1,185	496(301)	201(63)	30(7)

a Number of proteins identified in soluble (outside brackets) and membrane (within brackets) fractions of the proteome that have passed the filtering criteria.

b Number of protein identifications with high confidence in membrane, soluble and total fractions of the proteome.

c Analysis does not include the number of differentially expressed undefined intergenic regions in the transcriptome.

d Number of up-regulated (outside brackets) and down-regulated (within brackets) genes and proteins.

e Number of genes and proteins significantly up- or down-regulated in both the transcriptomic and proteomic analysis.

e NA; not applicable.

The procedures used for microarray and 2D-LC/MS/MS analysis, were as previously described by Kocharunchitt et al. [11].

### Analysis of microarray data

Gene expression was analysed as previously described [11]. Briefly, array data was normalized using the Robust Multi-Array Analysis (RMA) algorithm [17] in GeneSpring GX 10.0 software (Agilent Technologies, Palo Alto, CA). Probesets with an intensity value in the lowest 20th percentile among all the intensity values were excluded and the remaining probesets were used for downstream statistical analysis. Significance was analysed using one way analysis of variance (ANOVA) using the multiple correction testing method of Benjamini and Hochberg [18] with a *P*-value cut-off of ≤0.01. Genes and proteins were considered to be differentially expressed only if the fold change was ≥2. However, it should be noted that 2-fold changes can also be biologically significant [19], [20].

### Analysis of MS/MS data

MS/MS spectra obtained from each protein sample of membrane and soluble fractions were managed by the Computational Proteomics Analysis System (CPAS), a web-based system built on the LabKey Server (v9.1, released 02.04.2009) [21]. The mass spectra produced were subjected to a semi-tryptic search against the combined databases of *E. coli* O157∶H7 Sakai (5,318 entries in total) downloaded from the National Center for Biotechnology Information (NCBI, https://www.ncbi.nlm.nih.gov/, downloaded 25.11.2008) using X!Tandem v2007.07.01 [22]. These databases included the *E. coli* O157∶H7 Sakai database (5230 entries, NC_002695.fasta) and two *E. coli* O157∶H7 Sakai plasmid databases, plasmid pO157 (85 entries, NC_002128.fasta) and plasmid pOSAK1 (three entries, NC_002127.fasta). The search parameters used were as follows: mass tolerance for precursor and fragment ions: 10 ppm and 0.5 Da, respectively; fixed modification: cysteine carbamidomethylation (+57 Da); and no variable modifications. The search results were then analyzed using the PeptideProphet and ProteinProphet algorithms from the Trans Proteomic Pipeline v3.4.2 [23], [24]. All peptide and protein identifications were accepted at PeptideProphet and ProteinProphet of ≥0.9, corresponding to a theoretical error rate of ≤2% [23], [25]. In addition, the false-positive discovery rate for each dataset was further assessed by searching the MS/MS spectra against the target-reversed (decoy) database with the same search criteria as described above. The peptide false-positive discovery rate for each proteomic dataset was less than 5% for all MudPIT runs.

All protein identifications that met the above criteria were further assessed based on the confidence level of protein identifications across biological replicates of each fraction of *E. coli* at each time point. The confidence level was based on the number of unique peptides identified from one sample and the number of replicates in which the protein was detected. Specifically, proteins identified by more than one unique peptide in at least one of the replicates were considered to have a ‘high’ confidence score. The ‘intermediate’ confidence level was assigned to proteins with a single peptide hit that was detected in more than one replicate. ‘Low-confidence’ proteins were considered to be those identified by a single unique peptide and found in only one replicate. Only protein identifications with a ‘high’ and ‘intermediate’ confidence level (referred to as having a ‘high confidence score’) were accepted for further analysis.

Following data filtering, all protein identifications with ‘high’ confidence from membrane and soluble fractions of the same sample were combined to represent a ‘total’ fraction of the corresponding sample. This combined approach was employed on the basis that the observed R^2^ values, as determined by linear regression analysis, indicated a relatively stronger linear correlation between spectral counts (SpCs, the number of MS/MS spectra) for all possible pair-wise comparisons of replicates of pooled fractions (average R^2^ = 0.89±0.05) when compared to that of replicates of each fraction (average R^2^ = 0.64±0.19, and 0.85±0.05 for membrane and soluble fractions, respectively). Each of the total fractions across the biological replicates was then used to generate the final list of proteins for each time point of sampling.

### Protein expression ratio and its significance

Spectral counts generated by 2D-LC/MS/MS analysis were used as a semi-quantitative measure of protein abundance [23], [26]. The total abundance of each protein in a sample was obtained by pooling spectral counts detected for a given protein from membrane and soluble fractions of the same sample (i.e. the same biological replicate). Each pooled spectral count was then normalized to obtain the normalized spectral abundance factor (NSAF) according to Zybailov et al. [27]. Briefly, the NSAF for a given protein is the pooled spectral count of the protein divided by its amino acid length (L), and normalized against the average SpC/L across all proteins in the corresponding dataset. The NSAF values were then averaged across all biological replicates at each time point of sampling to obtain representative values of NSAF.

Fold changes in protein abundance due to cold shock were calculated as the log_2_ ratio of the average NSAF value in the cold-treated sample over the average NSAF value of the control. Control samples (35°C) were pooled from the current and companion study [28]. To avoid errors during fold change calculation in cases where a zero value was obtained, a correction factor of 0.01 was added to each of the average NSAF values. Furthermore, statistical analysis of differences in protein abundance was carried out using the beta-binomial test implemented in R [29]. All proteins with a *P*-value ≤0.01 and at least a 2-fold change were considered to be differentially abundant.

### Transcriptomic and proteomic data mining

Information on identified genes and proteins such as protein and gene names, EC numbers (locus tag), GI numbers, NCBI Reference Sequence (RefSeq), protein sizes and molecular masses were obtained from the UniProt knowledgebase (http://www.uniprot.org/) and the NCBI database (accessed on 5.12.2010). Preliminary functions and properties of the genes and proteins were based on the EcoCyc database (http://www.ecocyc.org/, accessed on 13.12.2010), and the Kyoto Encyclopedia of Genes and Genomes (KEGG) database (http://www.genome.jp/kegg/, accessed on 4.12.2010). In the present study, protein or gene names in conjunction with EC numbers were used as a unique identifier for genes and/or proteins.

### Predefined group enrichment analysis

To identify changes in the overall expression of a predefined set of genes or proteins due to temperature downshift, the *t*-test based method of Boorsma et al. [30], known as T-profiler, was performed on the normalized log_2_ expression ratios. The predefined sets of genes and proteins used in the T-profiler analysis were based on the selected functional role categories and/or metabolic pathways of the following databases: the JCVI Comprehensive Microbial Resource (JCVI CMR) database (http://cmr.jcvi.org/cgi-bin/CMR/CmrHomePage.cgi, accessed on 5.12.2010), the EcoCyc database collection (http://www.ecocyc.org/, accessed on 13.12.2010), and the KEGG database (http://www.genome.jp/kegg/, accessed on 4.12.2010), as well as based on the lists of genes or proteins previously known to be positively controlled by major regulons, including CpxRA [31], RpoE [32], RpoH [33], RpoS [34], [35], [36], and the Rcs phosphorelay system [37]. A complete list of genes and proteins with their comprehensive annotations are given in Table S1 in File S1. The *T*-values obtained from the analysis were determined only for sets that contained at least five members, and their significance was established by using the associated two-tailed *P*-value. All predefined sets with a *P*-value less than 0.1 were considered to be statistically significant.

### Clustering analysis of the transcriptomic and proteomic data

Hierarchical clustering analysis (HCA) was performed on the *T-*values calculated for JCVI CMR functional categories within each transcriptomic and proteomic profile. The degree of similarity of the transcriptomic and proteomic profiles of *E. coli* during exposure to a sudden downshift in temperature to 14°C were also compared to the T-profiler results for the transcriptome and proteome of *E. coli* during steady-state growth at 14°C a_w_ 0.985 [11]. The degree of similarity was calculated with the Euclidean distance metric and complete linkage as a clustering method, using Cluster 3.0 software [38]. The clustering result was then visualized in TreeView version 1.1.3 [39].

### Validation of microarray data by qRT-PCR

Three genes that showed significant (*P* value ≤0.01) up-regulation or down-regulation in the microarray experiments were selected for analysis by quantitative real-time reverse transcription-PCR (qRT-PCR). Forward and reverse PCR primers for *nuoC*, *fiu* and *spy* were designed using Primer3 software [40]. As previously described, the 16S rRNA gene (*rrsA*) was also included for normalization within samples [41], [42]. The primer pairs were as follows. For *rrsA*: forward (F), 5′-AGGCCTTCGGGTTGTAAAGT-3′; reverse (R), 5′-GTTAGCCGGTGCTTCTTCTG-3′. For *nuoC*: forward (F), 5′-TTGACTTACACGGCATGGAC-3′; reverse (R), 5′-TACGTGCAGGTCGTTTTCTG-3′. For *fiu*: forward (F), 5′- AGTCTGGCAGCGGTAACAGT-3′; reverse (R), 5′-GGTGAGCAACAGGCGTTTAT-3′. For *spy*: forward (F), 5′-TGCCTCTACCCTGGCTCTT-3′; reverse (R), 5′-TCGGTCAGGTTCAGGTCTTT-3′. One microgram of purified total RNA from each of the three biological replicates used for microarray analysis was reverse transcribed using the iScript cDNA synthesis kit (Bio-Rad, Hercules, CA). Real-time PCR was performed on tenfold dilutions (from 10^−2^ to 10^−4^) of the template cDNA. qRT-PCR mixes contained a total volume of 20 µl consisting of 10 µl IQ SYBR green supermix (Bio-Rad), 1.6 µl diluted cDNA, 0.4 µl each forward and reverse primer (25 µM stock), and 7.6 µl nuclease-free water (Ambion, Austin, TX). All Real-time PCR reactions were performed on the LightCycler 480 (Roche) under the following conditions: 95°C for 3 min, 45 consecutive cycles consisting of 95°C for 10 s and 60°C for 30 s, and 72°C for 30 s. Melting curve analysis (55 to 95°C, increment of 0.11°C/sec) was performed to ensure PCR specificity. The method described by Pfaffl (66) was employed to determine the expression fold changes of the target gene in cultures at each time point post-temperature downshift, compared to before cold shock (i.e. the reference culture).

### Transcriptomic and proteomic data Accession Numbers

The transcriptomic data are available in the ArrayExpress database (www.ebi.ac.uk/arrayexpress) [43] under accession number E-MTAB-2010. The mass spectrometry proteomic data are deposited to the ProteomeXchange Consortium (http://proteomecentral.proteomexchange.org) via the PRIDE partner repository [44] with the dataset identifiers PXD000583 and PXD000573.

## Results and Discussion

### Growth of E. coli O157∶H7 Sakai upon temperature downshift

**Figure 1:**
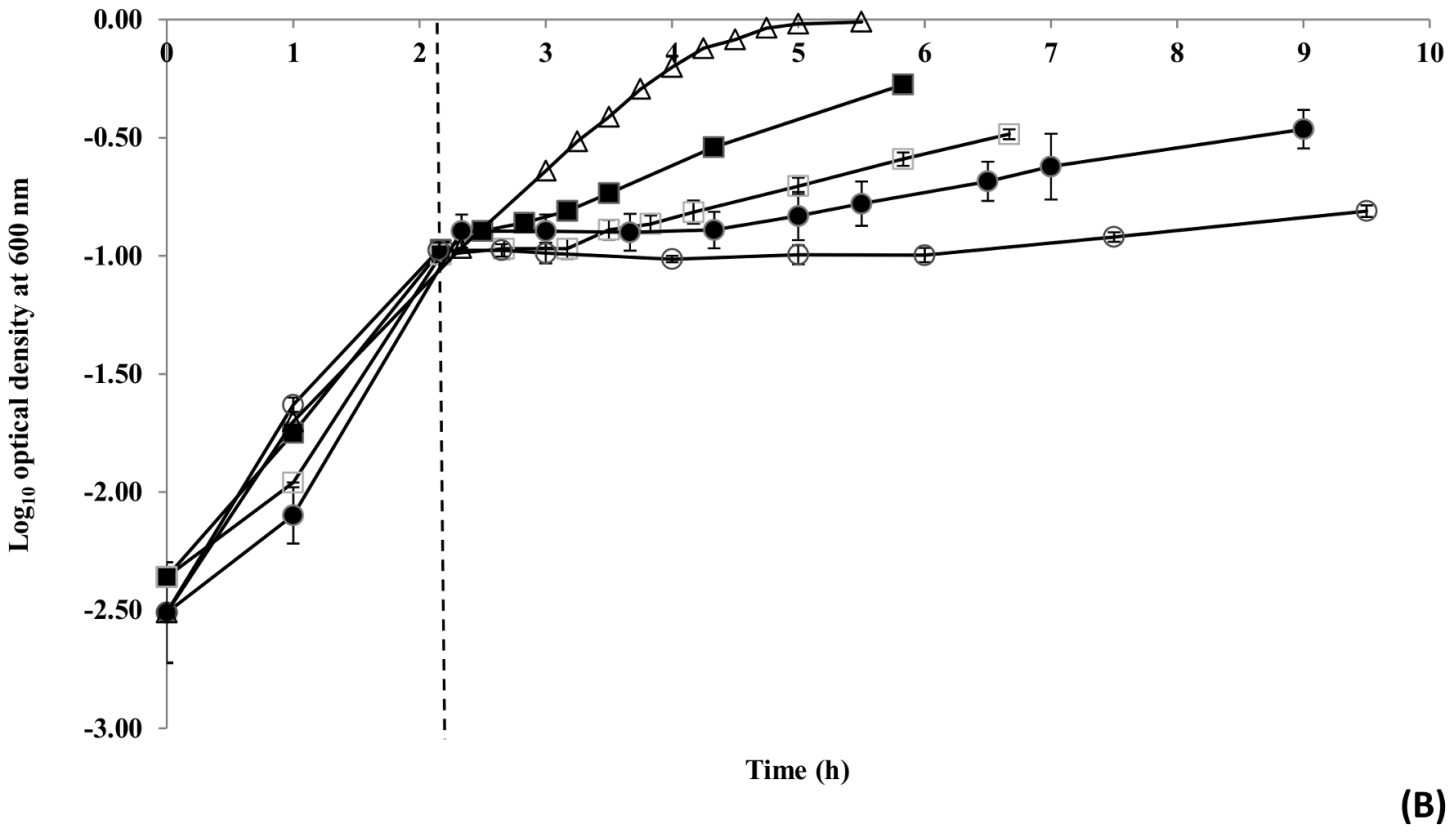
Growth response of exponential phase *E. coli* O157∶H7 Sakai to a rapid downshift in temperature. The growth response was monitored by measuring the optical density of cultures shifted from 35°C (Δ) to 20°C (▪), 17°C (□), 14°C (•) and 10°C (○). The time at which cold shock was applied is indicated by a dotted line. Data points represent means ± standard deviations of at least two independent replicates.

The growth response of *E. coli* upon temperature downshift of exponential phase cultures in a nutrient rich medium was examined. Specifically, cultures were exposed to a sudden downshift in temperature from 35°C to either 20°C, 17°C, 14°C or 10°C. It was found that all shifts induced a lag period before growth resumed at a slower rate than that before the treatment (Figure 1 and Table 2). The generation times observed at the post-downshift temperature were consistent with the predictive model of Ross et al. [45] and the lag time increased with the magnitude of the shift. However, the RLT response for *E. coli* showed no systematic variation across the temperature range tested, indicating that the amount of “work” the population had to perform to adjust to each temperature was similar (Table 2). This agrees well with Mellefont and Ross [15], who demonstrated that shifting *Klebsiella oxytoca* cells from a temperature within the normal physiological range to temperatures outside this range did not appear to have an effect on RLT. The normal physiological temperature range of *E. coli* is estimated to be from approximately 20°C to 38°C [15], [46], [47].

**Table 2:** The growth response of exponential phase *E. coli* O157∶H7 Sakai upon an abrupt temperature downshift from 35°C to either 20°C, 17°C, 14°C or 10°C; as determined by optical density.

Temperature downshift	Generation time (h)	Lag time (h)	Relative lag time (RLT)
**20°C**	1.29	0.30	0.23
**17°C**	1.93	0.84	0.44
**14°C**	3.28	1.95	0.59
**10°C**	9.01	3.81	0.42

### Molecular response of E. coli O157∶H7 Sakai to temperature downshift

**Figure 2:**
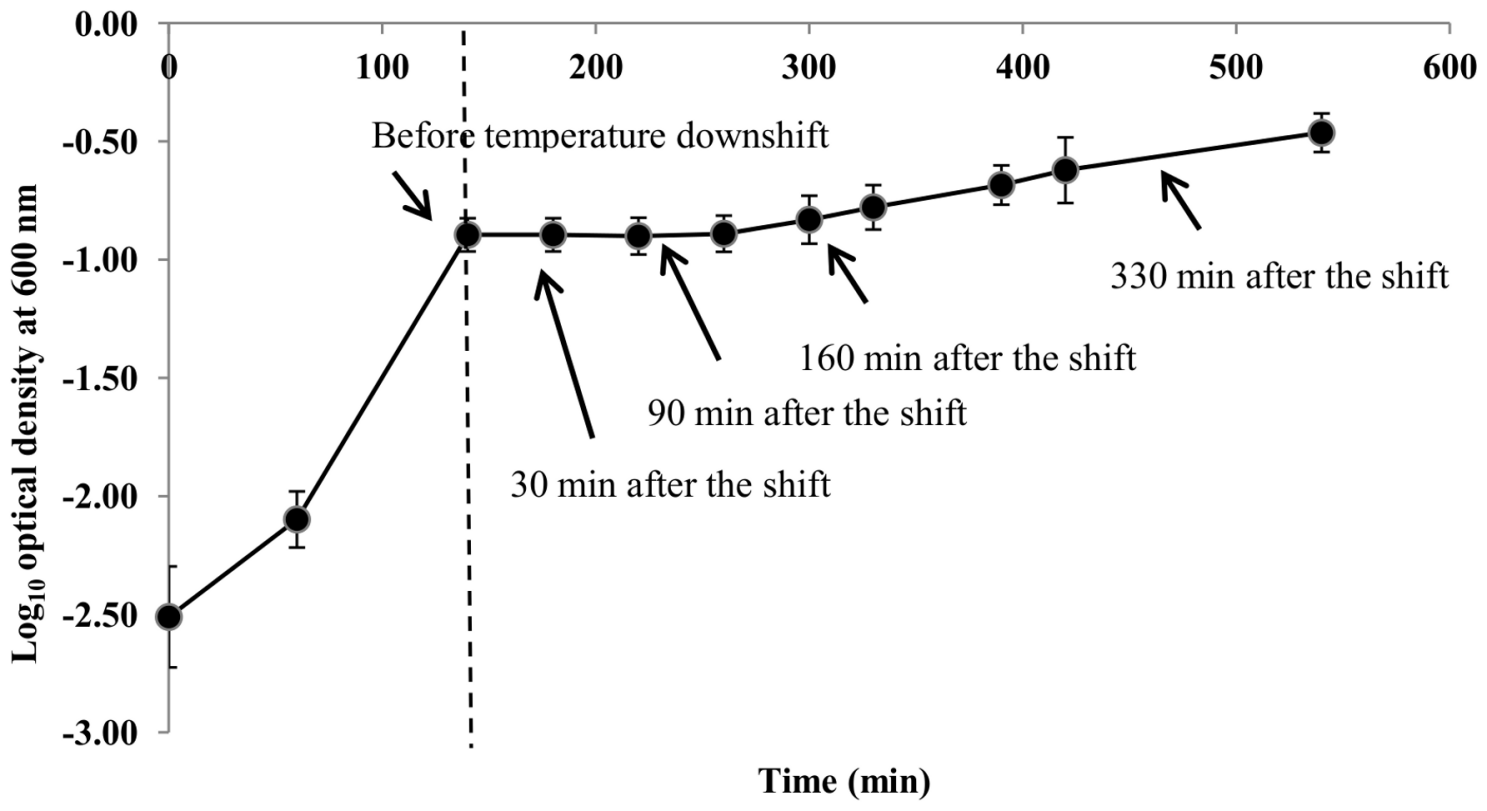
Time series of *E. coli* O157∶H7 Sakai during a temperature downshift from 35°C to 14°C. Solid arrows indicate the times at which samples were harvested for cDNA microarray and 2D-LC-MS/MS analyses. The time at which temperature downshift was applied is indicated by a dotted line. Data points represent means ± standard deviations of at least two independent replicates.

To reveal the entire genetic system that operates during the response of *E. coli* to an abrupt shift to chill temperature, we analysed the transcriptional and proteomic response of *E. coli* O157∶H7 Sakai during exponential growth at 35°C upon a shift to 14°C. Samples were taken for transcriptomic and proteomic analysis to determine temporal gene and protein expression profiles of cultures during cold adaptation (30 and 90 min after a temperature downshift) and in cold-adapted cells which had resumed exponential growth (160 and 330 min post-temperature downshift) (Figure 2). Independent samples were then processed for mRNA, and membrane and soluble proteins as appropriate for analysis by cDNA microarray and 2D-LC-MS/MS, respectively. Through multidimensional LC-MS/MS analysis together with data filtering, a number of protein identifications with high confidence were detected in total fractions of *E. coli* at each time point.

**Figure 3:**
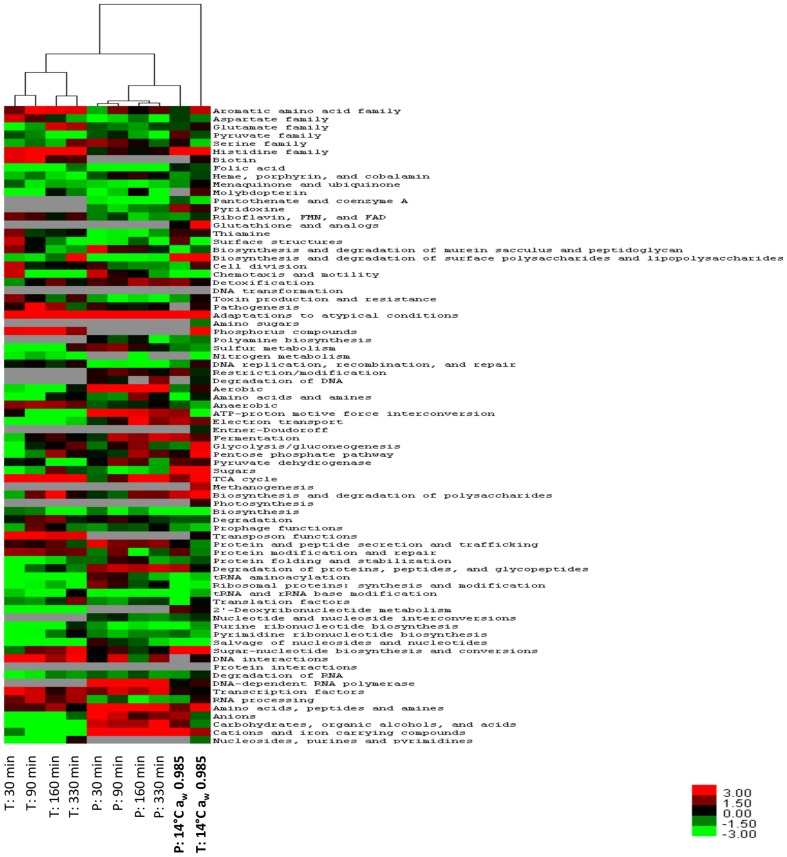
Hierarchical clustering analysis of the transcriptomic and proteomic data. The heat map represents the *T-*values calculated for the transcriptomic (T) and proteomic (P) profiles based on JCVI CMR functional categories. Within the dendrogram transcriptomic and proteomic profiles are clustered along with transcriptomic and proteomic profiles of exponentially growing cultures at 14°C a_w_ 0.985 [11]. Positive *T*-values are represented in red, negative *T*-values are represented in green and, grey indicates that the *T*-value has not been determined.

Gene and protein expression profiles at each of these time points were compared with those of a control culture at 35°C (i.e. reference sample). The number of differentially expressed genes and proteins at each time point are shown in Table 1. In the transcriptomic and proteomic analysis up-regulation was predominant over down-regulation at all time points, consistent with a previous time course study examining the genome-wide transcriptional alterations in *Vibrio parahaemolyticus* upon sudden temperature downshift [48]. A comprehensive list of the differentially expressed genes and proteins, as well as their log_2_expression ratio and a comparison to previously published data is given in Table S1 in File S1. A low level of correlation was observed between the transcriptomic and proteomic profiles generated at each time point, with between 0.4 and 6.1% of those genes significantly differentially expressed at each time point also significantly differentially expressed as proteins (Figure S1 in File S1). Indeed, clustering analysis of the T-profiler analysis of the transcriptomic and proteomic data revealed that the two datasets formed two discrete clusters (Figure 3). Within each dataset expression profiles clustered with time, clearly displaying a difference in the expression response during cold adaptation in lag phase (30 and 90 min) and the exponential phase of growth (160 and 330 min). The discordant alignment of transcript and protein levels is in keeping with findings from our previous study investigating the integrated transcriptomic and proteomic physiological response of *E. coli* Sakai to steady-state conditions relevant to low temperature and water activity conditions experienced during carcass chilling in Australia [11], as well as several other published reports [49], [50], [51], [52]. In light of the low level of correlation between the transcriptomic and proteomic datasets, qRT-PCR was employed to validate the microarray results of representative genes showing significant (*P* value ≤0.01) up-regulation or down-regulation. The results of the qRT-PCR experiments confirmed the microarray results (Table 3). Several possible explanations have been proposed for the low level of correlation between transcript and protein levels, including posttranslational regulation and differences in the turnover rate of mRNA versus the half-lives of encoded proteins. However, while there was not a high level of concordance in the genes and proteins differentially expressed (Table 1), a number of trends were observed in the expression patterns of predefined functional categories of genes/proteins and metabolic pathways (Figure 3). Thus while the transcriptomic and proteomic data give a broad and consistent view of the changes occurring, discrepancies exist at the micro level and the reasons for this remain to be determined.

**Table 3:** Gene expression fold changes resulting from microarray analysis and qRT-PCR for three selected genes.

Time (min after the shift)	Gene	Microarray fold change^a^	qRT-PCR fold change
**30**	*nuoC*	−2.29	−1.55±0.17
	*fiu*	NDE^b^	1.00±0.20
	*spy*	2.05	2.20±0.15
**90**	*nuoC*	−3.20	−3.33±0.09
	*fiu*	−6.75	−3.50±0.14
	*spy*	4.12	4.45±0.31
**160**	*nuoC*	NDE	1.31±0.28
	*fiu*	−9.84	−3.30±0.05
	*spy*	4.00	4.60±0.32
**330**	*nuoC*	NDE	1.04±0.12
	*fiu*	−2.60	−2.61±0.67
	*spy*	4.52	2.62±0.49

a Microarray results are expressed in fold change for ease of comparison.

b NDE, not differentially expressed.

We observed the universal up-regulation of five genes and proteins at every time point. Within the CspA family (from CspA-CspI); CspA, CspB, and CspG are termed the major cold shock proteins in *E. coli* on the basis of their levels of induction [53]. In our study *cspG* and CspG were universally up-regulated at every time point by an average of 5.09- and 6.62-log_2_ fold, respectively. Increased production of CspG has been reported to be essential for bacterial cells to resume growth at low temperature [54], [55]. However, our findings are inconsistent with a previous report that CspG is up-regulated during the acclimation phase upon cold shock and subsequently down-regulated when cells have become cold-adapted [56]. This discrepancy may be due to strain variation in the response of the O157∶H7 strain used in this study and the K-12 strain in the previously published report [56]. Of the remaining cold shock proteins we observed up-regulation of *cspA* (30, 90, 330 min) and CspA (90 mins onwards), *cspC* (330 min) and *cspH* (30, 90, 160, 330 min). While CspG may have overlapping functions with CspA, it is speculated that CspG may have specific function(s), as it is induced within a narrower low temperature range compared to CspA [57]. It is thought [57] that CspG may be required for transcription and/or DNA replication as a DNA chaperone. In our study, from the level of induction of CspG, it appears that this protein fulfils a unique role in enabling adaptation and growth of *E. coli* at 14°C. Amongst the other genes and proteins universally up-regulated are those previously reported to be cold-induced including an ATP-dependent helicase (*csdA*/CsdA) [58] and exoribonuclease R (*rnr*/Rnr) [59]. Phosphoethanolamine transferase (*eptB*/EptB) was also universally up-regulated and may have role(s) in envelope stress, as its expression is activated by RpoE [32], [60], [61]. The continuous up-regulation of these transcripts and proteins across the entire time-course indicates their importance beyond the initial phase of the acclimation period. Surprisingly, we also observed the persistent up-regulation of the universal stress protein (*uspE*/UspE). While cold shock has previously been shown to repress UspACDE expression [62], in this study UspE is suggested to have an important role during growth at low temperature. In the current study UspA (30, 90, 160, 330 min) and *uspC* (30, 90, 330 min) were also up-regulated (Table S1 in File S1). Strain variation between the K-12 MC4100 strain used in the previous study [62] and the O157∶H7 strain used in the current study, may account for this difference in response.

The cluster analysis (Figure 3) did not reveal a close similarity between the expression response profiles elicited by *E. coli* during exponential phase growth (160 and 330 min) and the expression response observed in our previous study monitoring the growth of *E. coli* during exponential growth at the same temperature (14°C a_w_ 0.985) [11]. This may be accounted for, in part, by the difference in water activity in the current study (a_w_ 0.993) and the previous study (a_w_ 0.985) [11]. However, broad similarities were revealed with all proteomic expression profiles across all time points clustering (Figure 3) with the proteomic response of *E. coli* during exponential growth at 14°C a_w_ 0.985 [11].

### DNA metabolism

The requirement to conserve energy is an important feature of all stress responses [36], [63], [64]. The implementation of the RpoS-dependent general stress response has previously been shown to reduce energy expenditure through the repression of genes involved in growth, cell division, and protein synthesis [36]. The T-profiler analysis revealed significant down-regulation of proteins involved in DNA replication, recombination, and repair at all time points, except at 90 min after imposition of the cold stress (Table S2 in File S1). In keeping with previous studies we also observed a greater than 1-log_2_ fold down-regulation in the expression of a number of proteins involved in these functions after 30 (TopA, MutS), 90 (MutS), 160 (TopA, StpA, MutS) and 330 (MutS) min of the imposition of cold stress [10], [65], [66]. Interestingly, the DNA mismatch repair gene/protein *mutS*/MutS was down-regulated at every time point and was amongst the 20 most highly down-regulated proteins at 30 and 160 min. MutS expression decreases in stationary phase and this pattern of regulation has been proposed to reflect a mechanism in which cells coordinate the amount of MutS with decreases in the level of DNA replication [67]. The reduction in MutS expression reflects the changes in the expression of genes and proteins involved in transcription and translation. Interestingly, it has previously been suggested [68] that down-regulation of MutS may contribute significantly to increased mutation or homologous recombination within populations in stressed environments. We did not, however, observe increased expression of other genes and proteins of the very short patch (VSR) mismatch repair system, to compensate for down-regulation of MutS (Table S1 in File S1). Likewise, increased expression of *mug*/Mug and *mutY*/MutY was not observed (Table S1 in File S1).

### Transcription and translation

As the incubation temperature after temperature shift decreased, the corresponding growth rate decreased, and so should the rate of protein synthesis, DNA replication and the need for nucleotides. In addition, one of the major physiological effects of cold temperature is the stabilization of secondary structures of RNA and DNA, which may affect the efficiencies of translation, transcription, and replication [69]. In keeping with this, we observed negative *T*-values for genes involved in tRNA aminoacylation at every time point, genes (90 and 160 min) and proteins (90, 160 and 330 min) involved in tRNA and rRNA base modification, genes involved in the synthesis and modification of ribosomal proteins (30 and 330 min), genes involved in 2' deoxyribonucleotide metabolism (30, 90, 160 and 330 min), genes involved in the salvage of nucleosides and nucleotides (30, 90, 160 and 330 min) and, genes (30, 90 and 160 min) and proteins (90 min) involved in purine ribonucleotide biosynthesis (Table S2 in File S1). In keeping with the down-regulation of genes and proteins involved in purine ribonucleotide biosynthesis, we also observed down-regulation of genes (30, 90, 160 min) and proteins (30, 160 and 330 min) involved in folic acid biosynthesis (Table S2 in File S1). Folates are essential cofactors that facilitate the transfer of one-carbon units from donor molecules into important biosynthetic pathways leading to methionine, purine, and pyrimidine biosynthesis.

### Carbohydrate catabolism and energy generation

Genes involved in glycolysis (30 and 90 min), gluconeogenesis (30 min), the pentose phosphate pathway (30 min), mixed acid fermentation (30 min), and oxidative phosphorylation (30, 90 and 160 min) were down-regulated (Table S3 in File S1). In contrast to the transcriptomic results, the proteomic data revealed significant increased abundance of proteins involved in oxidative phosphorylation at every time point, indicating a perturbation in respiratory balance (Table S3 in File S1). Indeed a previous report on the transcriptomic and proteomic response of *Bacillus subtilis* to growth at low temperature, reports a reduction in oxidative phosphorylation in line with a reduced energy demand during slow growth at low temperature [70].

An initial general reduction in the expression of genes involved in carbohydrate catabolism also parallels the down-regulation of sugar transporters (see below).

TCA cycle and glyoxylate bypass genes were up-regulated at every time point and this was mirrored in the proteomic data at 160 and 330 min (Table S3 in File S1). This corresponds with previous findings, in which temperature downshift resulted in increased expression levels of genes encoding enzymes in the TCA cycle to compensate for the reduction in activity of TCA cycle enzymes at lower temperatures [71]. However, oxygen consumption rates have also been shown to be lower at lower temperatures and it has been suggested that this points towards a reduced carbon flux through the TCA cycle and a reduced electron flux through the electron transport pathway [71]. In the current study, the T-profiler analysis of the transcriptomic study also revealed down-regulation of electron transport-associated genes at 30 mins (*ykgC*, *yddW*, *dusC*, *ccmE*, *nuoBCF*, *hmp*, *uxuB*), 90 mins (*kefF*, *ykgC*, *cydAB*, *trxB*, *yddW*, *sufBD*, *yedF*, *yeeD*, *dusC*, *ccmCEFH*, *napGB*, *yfaE*, *nuoABCEFHIJKLM*, *hmp*) and 160 mins (*cydAB*, *cydC*, *nuoHIJKLMN*, *ccmCEFH, trxB*, *ndh*, *yddW*, *yeeD*, *dusC*, *napC*, *yfaE*, *hmp*, *yigI*, *ycaO*). Real-time PCR was used to validate these results and down-regulation of *nuoC* was also observed at 30 and 90 mins (Table 3).

### Amino acid biosynthesis

The aspartate and pyruvate biosynthetic pathways were negatively regulated after a shift to 14°C (Table S2 in File S1), correlating with a previous report on the transcriptional response of *E. coli* to temperature downshift [71]. In addition, genes encoding tRNA synthetases were down-regulated at 30 mins (*aspS*, *argS*, *gltX*, *lysS*, *glyS*), 90 mins (*tyrS*, *pheS*, *aspS*, *argS*, *gltX*, *alaS*, *lysS*, *trpS*, *yadB*), 160 mins (*pheST*, *argS*, *gltX*, *lysS*, *lysU*, *yadB*) and 330 mins (*lysU*, *yadB*). Analysis of the proteome only revealed significant down-regulation of prolyl-tRNA synthetase (ProS) at 330 min. As has previously been suggested in a transcriptional analysis of the cold shock response of *B. subtilis* [65], in the current study the slower growth rate induced after a shift to 14°C resulted in a general reduction in protein synthesis which may lead to an oversupply of amino acids and feedback inhibition of genes associated with amino acid biosynthesis (Table S2 in File S1).

Activation of specific amino acid biosynthesis pathways or amino acid supplementation can lead to tolerance to certain environmental stresses [72]. In a report examining the thermal adaptation of *Exiguobacterium sibiricum*, a psychrotrophic bacterium isolated from 3 million year old Siberian permafrost that grows from −5°C to 39°C, it was demonstrated that cold stress leads to flux and pool size redistribution throughout the entire network of amino acid metabolism [73]. Rodrigues et al. [73] suggested that the reason for a change in cell metabolism may be to synthesize more ‘flexible’ proteins that can function at lower temperatures. In the current study, the positive regulation of specific amino acid biosynthesis pathways was in keeping with previous reports of the response of *E. coli* to chill temperature with positive *T*-values for the histidine family (90 and 330 min) [11], [63] and positive *T*-values for amino acid biosynthesis in the aromatic amino acid family at 90 min with the significant up-regulation of genes involved in the biosynthesis of phenylalanine (*pheA*, *aroG*), tyrosine (*pheA*), tryptophan (*trpEDCBA*) and chorismate (*aroL*, *aroE*) [11]. Deciphering the role histidine and aromatic amino acids play under these conditions would provide insight into whether the synthesis of these amino acids is vital for adaptation and growth at chill temperature.

### Central intermediary metabolism

We observed negative *T*-values and greater than a 1-log_2_ fold down-regulation of genes involved in sulfur metabolism at 30 mins (*sseA*, *cysI*, *cysDN*), 90 mins and 160 mins (*cysDNC*, *cysI*) (Table S2 in File S1). This appears related to reduction in the synthesis of the sulfur containing amino acids; such as cysteine, as we also noted down-regulation of *cysK* (30 and 90 min) encoding the cysteine synthase A which carries out the second step in the pathway of cysteine biosynthesis. However, this contradicts previous findings of cold shock induction of *cysK* in *E. coli* at 15°C [10]. Strain variation between the K-12 JM83 strain used in the previous study [10] and the O157∶H7 strain used in the current study, may account for this difference in response.

### Transport and binding proteins

Cold-induced remodelling of the bacterial cell envelope can affect the transport/binding functions that are essential for the accumulation of nutrients against a concentration gradient, as well as for the excretion of various end products of metabolism [48]. Cold shock has been shown to result in extensive alterations of the cell envelope structure and transmembrane transporting functions [48]. In keeping with this, we observed numerous changes in expression patterns of genes and proteins involved in transport and binding (Table S2 in File S1). Of interest was an increase in the expression pattern at all time points of proteins involved in the transport of amino acids, peptides and amines (Table S2 in File S1). We also observed greater than a 2-log_2_ fold induction of proteins involved in the transport of iron, the osmoprotectants proline and glycine betaine, and uncharacterised substrates at 30 mins (FhuC, YojI), 90 mins (FhuC, YojI, YadG, ProV), 160 mins (FhuC, YojI, ProVW) and 330 mins (FhuC, YojI, ProVW, YadG, ProP, YehZ). The chill stress response in bacteria includes the uptake and synthesis of compatible solutes [74], [75], [76] and proline transporters have previously been shown to be up-regulated upon exposure of *E. coli* to reduced temperature [71].

A contrasting expression pattern was observed for genes and proteins involved in the transport of cations and iron-carrying compounds, with the down-regulation of genes (90, 160 and 330 min) and up-regulation of proteins (30, 90, 160 and 330 min) (Table S2 in File S1). The proteomic data revealed greater than a 2-log_2_ fold increase in expression of a putative outer membrane receptor for iron transport (Fiu) and a magnesium ion transporter (CorA) at every time point, while the transcriptomic data displayed greater than a 1-log_2_ fold down-regulation of *fiu* (90, 160 and 330 min) and *corA* (90 min). Real-time PCR was employed to validate these results and down-regulation of *fiu* was also observed at 90, 160 and 330 min (Table 3). The reason for the significant difference in the expression pattern of these elements in the transciptome and proteome remains to be determined.

The transcriptomic data also revealed decreased expression of genes involved in anion transport at 30, 90 and 160 min (Table S2 in File S1). Genes involved in the transport of sulfate, gluconate, inorganic phosphate and arsenite were down-regulated by greater than 1-log_2_ fold at 30 mins (*pstSCA*, *cysPUWA*, *cysZ*, *gntU*), 90 mins (*arsB*, *pstSCAB*, *cysPUWA*, *cysZ*, *gntU*), 160 mins (*arsB*, *pstSCAB*, *cysPUWA*, *gntU*) and 330 mins (*arsB*, *pstSCA*).

Negative regulation was observed in the T-profiler analysis for genes involved in the transport of carbohydrates, organic alcohols and acids at 30, 90 and 330 min (Table S2 in File S1). The majority of those genes with greater than a 1-log_2_ fold reduction in expression at 30 mins (*manXYZ*, *malE*, *treB*, *uhpA*, *ptsG*, *ptsHI*, *gntU*, *yhjX*, *mtlA*, *mglA*), 90 mins (*manXYZ*, *malE*, *treB*, *uhpABC*, *ptsG*, *ptsHI*, *gntU*, *yhjX*, *mtlA*, *focA*, *etk*, *fadL*, *rbsA*), 160 mins (*manXYZ*, *treB*, *uhpABC*, *ptsG*, *ptsHI*, *gntU*, *mtlA*, *dcuA*, *fadL*, *rbsA*) and 330 mins (*manX*, *malE*, *treB*, *uhpABC*, *uhpT*, *malF*, *dcuA*) were involved in the transport of sugar (i.e. mannose, maltose, trehalose, glucose, galactose, ribose). The down-regulation of these sugar transport genes coincides with the general down-regulation of genes involved in carbohydrate catabolism (Table S2 in File S1).

Genes involved in the transport of nucleosides, purines and pyrimidines were down-regulated at 30 and 90 min (Table S2 in File S1). However Tsx, which is involved in the high affinity uptake of ribo- and deoxy-nucleosides across the outer membrane, was amongst the 20 most highly up-regulated proteins at every time point.

### Phospholipid and fatty acid metabolism

Exposure to cold shock or growth at low temperature has effects on the outer membrane lipid composition that are beneficial to the cell [5]. Palmitoleate is not present in lipid A isolated from *E. coli* cells grown above 30°C, but has been shown to comprise ∼11% of the fatty acid acyl chains of lipid A in cells grown at 12°C [77]. While not observed in the proteomic analysis, in the current study the palmitoleoyl transferase encoding gene *lpxP* displayed an average increase in expression of 3.60-log_2_ fold across all time points.

A decrease in growth temperature leads to an increased proportion of unsaturated fatty-acid residues in microbial lipids, which results in a lowering of the melting point of the lipids and preserves membrane fluidity that is necessary for survival and growth [5]. However, in the current study the pattern of expression of genes and proteins involved in fatty acid metabolism was not significantly affected upon temperature shift (Table S2 and S3 in File S1). The ratio of saturated to unsaturated fatty acids is controlled by the relative levels of FabA and FabB, with overproduction of FabA leading to an increase in synthesis of saturated fatty acids [78]. In keeping with a previous report [71], the expression level of *fabA*/FabA remained unchanged after temperature shift for the entire time course. Contrasting with that report [71] we did not observe a significant increase in expression of *fabB*/FabB. However, *sfa* displayed an average increase in expression of 3.22-log_2_ fold across every time point. While not observed in our proteomic data, expression of Sfa results in increased biosynthesis of unsaturated fatty acids, presumably by increasing the activity of FabB [79].

Diffusion rates are reduced at low temperature and the outer membrane diffusion pore protein, OmpF, is expressed at an elevated level in the cold compared to optimal growth temperature [8]. At the same time the amount of OmpC is reduced [8]. In our study *ompF*/OmpF was up-regulated at 330 min (Table S1 in File S1). However, we also observed up-regulation of OmpC at 90 and 330 min (Table S1 in File S1).

### Bacterial chemotaxis and motility

Genes involved in chemotaxis and motility were significantly down-regulated, as revealed by significant negative *T*-values at 90, 160 and 330 min (Table S2 and S3 in File S1). This coincides with our previous findings, that chemotaxis and motility genes and proteins are down-regulated during balanced growth at 14°C [11]. The down-regulation of flagellar genes has also been reported in *E. coli* during shift to incubation temperatures of 15°C [80] and 16°C [63], as well as in *B. subtilis* during growth at low temperature [70]. Expression of genes encoding flagella is negatively controlled by the master regulator RpoS [35] and we also observed an average 1.33-log_2_ fold increase in expression of *rpoS* from 90 min onwards. However, these findings are at odds with the general increase in expression of *uspE*/UspE and its essential role in motility [81] and may suggest another physiological role for UspE during adaptation and growth at 14°C. Indeed, UspE has previously been suggested to play a crucial role in oxidative stress resistance [81].

The down-regulation of chemotaxis and motility genes in the current study may be due to energy deficiency, consistent with a previous study [63] proposing that decreased cell motion could indicate energy deficiency as flagella motility requires a steep proton gradient between the periplasmic space and the cytoplasm. This is inconsistent with the observation of significant up-regulation of several methyl-accepting chemotaxis proteins (MCPs); which are involved in sensing chemical attractants that are sources of carbon and energy. *E. coli* possesses four MCPs and we observed significant up-regulation of Tar (30 min), Tsr (30, 90 and 160 min) and Tap (90, 160 and 330 min) (Table S1 in File S1). Tar mediates taxis toward aspartate and maltose, while the primary attractant detected by Tsr is serine and Tap is required for taxis toward dipeptides and pyrimidines [82]. The up-regulation of Tar, Tsr and Tap contradicts previous reports that their genes are negatively regulated by RpoS [83]. This finding suggests other regulatory factors may override the control of RpoS over Tar, Tsr and Tap.

### Major regulons and stress responses

The transcriptomic data revealed an initial increase in the expression of transcription factors at 30 min (Table S2 in File S1), with eight genes encoding transcription factors showing greater than a 1-log_2_ fold increase in expression. These included a number of major stress response sigma factors (*rpoE*, *rpoN*, *rpoH*).

Genes and proteins of the RpoS regulon [34], [35], [36] were up-regulated from 90 min onwards (Table S4 in File S1). This is consistent with low temperature induction of RpoS under steady state conditions previously reported [11], [84]. Nineteen genes exhibited greater than a 1-log_2_ fold increase in expression from 90 min onwards and included genes involved in the osmotic stress response (*osmY*), acid response (*gadX*), biofilm formation (*bolA*), cell wall biogenesis (*erfK*), encoding an inner membrane protein (*yqjE*), predicted outer membrane protein (*ybaY*), hypothetical proteins (*yahO*, *yaiA*, *yccJ*, *ydiZ*, *yodC*, *yqjCD*, *yhhA*, *ytfK*, *ygaM*) and putative and predicted transcriptional regulators (*yiaG*, *yjbJ*, *nsrR*). In contrast, only two proteins of the RpoS regulon displayed greater than a 1-log_2_ fold increase in expression from 90 min onwards including an osmotic stress response protein (OsmY) and a putative outer membrane protein (YeaY).

The RpoE extracytoplasmic stress response regulon was initially up-regulated, as revealed by a positive *T*-value in the transcriptomic data at 30 min (Table S4 in File S1). The *rpoE* gene encoding the RpoE sigma factor displayed an average increase in expression of 1.97-log_2_ fold across the 30 and 90 min time points. Of those genes of the RpoE regulon [32] 16 were up-regulated at 30 min including *rpoE* and its regulators (*rseABC*); the nitrogen-related (*rpoN*) and heat shock (*rpoH*) sigma factors; genes associated with lipid A biosynthesis (*ddg*), zinc export (*zntR*), asparagine metabolism (*ansB*) and phosphopantetheinyl transfer (*yieE*); a stress-inducible periplasmic protease (*degP*); a helicase (*lhr*); regulators involved with pyrimidine metabolism, purine degradation and pH homeostasis (*rutR*); fatty acid biosynthesis (*fabR*), and a gene encoding an outer membrane protein (*yidQ*). We also noted that amongst the 20 most highly up-regulated proteins at all time points was a putative outer membrane lipoprotein of the RpoE regulon (YeaY). In keeping with this, Polissi et al. [69] reported that *rpoE* exhibited an early transient increase in mRNA abundance 1 h after cold shock at 16°C. RpoE is believed to be activated by, and to respond to, misfolded proteins in either the periplasm or outer membrane [85]. However, it remains to be determined how the RpoE regulatory pathway is involved in low temperature adaptation in response to cold induced extracytoplasmic stress [69].

The *rpoH* gene was up-regulated across all time points by an average of 1.68-log_2_ fold. However, genes (30 and 90 min) and proteins (30 min) of the RpoH regulon [33] were down-regulated (Table S4 in File S1). At 30 and 90 min, seven genes encoding heat shock chaperones (*dnaKJ*, *htpG*, *ppiB*, *grpE*, *groEL*) were down-regulated by over 1-log_2_ fold. Genes and proteins of the RpoH regulon which were commonly down-regulated included *htpG* (30, 90 and 330 min) and HtpG (330 min), and *prlC* (90 min) and PrlC (330 min). This is not surprising in light of the role of HtpG as a heat shock chaperone and the fact that PrlC is believed to be involved in the onset of DNA replication [86]; for which we see a general down-regulation in the proteomic data (30, 160 and 330 min; Table S2 in File S1). While it has previously been observed that genes encoding heat shock-inducible proteins and molecular chaperones (*htpG*, *groEL*, *groES*, *ppiA*) were up-regulated following cold shock, this induction was transient [10]. Interestingly, it has previously been shown that the growth arrest of *E. coli* upon cold shock appeared to be associated with inactivation of the GroELS chaperone system [87].

Genes of the CpxRA regulon, which are involved in the envelope stress response and in the control of the biogenesis of adhesive structures such as pili [31], were up-regulated at every time point (Table S4 in File S1). However, this was not mirrored in the proteomic data. We observed the up-regulation of three genes by over 1-log_2_ fold at every time point, including genes involved in spheroblast formation (*spy*), regulation of the extracytoplasmic stress response (*yihE*; [88]) and peptidoglyan biosynthesis (*ycfS*). Real-time PCR was used to validate these results and up-regulation of *spy* was also observed at every time point (Table 3). The Cpx system also displayed greater than a 1-log_2_ fold increase in expression (*cpxR*; 90, 160 min, *cpxA*/CpxA; 90, 160, 330 min). Other signs of envelope stress were indicated by the greater than 1-log_2_ fold induction of *degP*, encoding a stress-inducible periplasmic protease (30, 90, 160 min). In line with the general down-regulation of chemotaxis and motility genes (Table S2 and S3 in File S1), *ydeH* was up-regulated an average of 1.94-log_2_ fold from 90 min onwards. The *ydeH* gene encodes a diguanylate cyclase that regulates motility and biofilm formation and represses swimming behaviour, reduces the abundance of flagella and abolishes the appearance of pili-like structures [89].

The T-profiler analysis revealed a significant positive *T*-value for the Rcs regulon [37], which controls the biosynthesis of extracellular polysaccharide capsule or colanic acid, in the transcriptomic study at 160 and 330 min (Table S4 in File S1). A number of genes and proteins involved in colanic acid biosynthesis had at least a 1-log_2_ fold increase in expression at 30 (*ugd*) and 330 (*ugd*, *cpsB*, *gmd*, *wcaG*, GalK) min. Genes and proteins known to be induced by the Rcs regulon were amongst the most highly up-regulated under all steady-state conditions in our previous study [11]. The present findings are also consistent with the report of Hagiwara et al. [37] concerning induction at low temperatures.

The RpoS-dependent *otsA*/*B* genes were also up-regulated on average by 1.99-log_2_ fold in the transcriptomic study at 160 and 330 min (Table S1 in File S1). This is in keeping with the induction of *otsA*/*otsB* and activation of trehalose synthesis during cold shock reported by Kandror et al. [90] and Moen et al. (49). Results from our steady state study [11] also revealed an up-regulation in the expression of trehalose biosynthetic enzymes (*otsAB*/OtsAB) under all conditions at 14°C. As a compatible solute, trehalose is thought to enhance cell viability when the temperature is reduced to near freezing [7], [90].

We observed a number of genes within the Acid Fitness Island (AFI) up-regulated by greater than l-log_2_ fold at 30 mins (*hdeD*, *gadE*, *gadX*), 90 mins (*hdeD*, *gadE*, *gadX*), 160 mins (*hdeD*, *gadE*, *gadX*, *dctR*, *yhiD*, *hdeB*) and 330 mins (*dctR*, *hdeB*, *gadE*, *gadX*). While we did not see enhanced representation of AFI proteins expressed across each of the time points, the “starvation lipoprotein” Slp was up-regulated by 3.46- and 5.05-log_2_ fold at 90 and 330 min, respectively. This suggests, as discussed earlier, that growth at 14°C may result in energy deficiency. We previously observed significant up-regulation of glutamate decarboxylase genes during exposure to cold stress under steady state conditions [11], as have others [9], [80]. In addition, genes of the glutamate-dependent *gad* system were up-regulated by greater than 1-log_2_ fold under all steady state conditions at 14°C in our previous study [11]. Polyamines (e.g., putrescine and spermidine) are known to play an important role during acid stress [91], and in the present study the transcriptomic data revealed up-regulation of genes of the *opp* operon (*oppABCDF*) that encodes a polyamine-induced oligopeptide ABC transport system at 90 min (*oppACDF*) and 160 min (*oppAC*). While the proteomic study revealed up-regulation at 90 min (OppB) and 330 min (OppBD).

Interestingly, amongst the 20 most highly down-regulated proteins was hydroperoxidase I (KatG) at 90, 160 and 330 min. A number of genes previously reported to be amongst the most highly up-regulated in response to exposure to oxidative stress were also amongst those down-regulated at 30 mins (*dps*, *grxA*, *cysK*, *ibpA*, *katG*), 90 mins (*yaaA*, *ahpF*, *dps*, *grxA*, *sufABC*, *yeeD*, *cysK*, *ibpA*, *katG*), 160 mins (*ahpF*, *grxA*, *sufC*, *yeeD*, *ibpA*, *katG*) and 330 mins (*grxA*, *sufC*, *ibpA*, *katG*) [92]. Indeed, in other studies cold-shocked *E. coli* and *Salmonella typhimurium* were reported to be sensitised to oxidative stress [93].

While we did not see significant up-regulation of *relA*/RelA or *spoT*/SpoT across the time course, the transcriptomic data displayed some of the hallmarks of a response mediated by ppGpp. In keeping with the stringent response elicited by *E. coli* in the transcriptomic study by Durfee et al. [64], we observed up-regulation of the RpoS, RpoE and RpoH regulons and the histidine biosynthesis pathway, and the down-regulation of such functions as protein synthesis, glycolysis, gluconeogenesis, respiration, motility and chemotaxis. This is not surprising, given that a reduced growth rate is a consequence of the imposed stresses.

## Conclusions

This study offers an empirical understanding of the growth kinetics and molecular response of exponential phase *E. coli* O157∶H7 Sakai to an abrupt temperature downshift. Shifting cells from 35°C to a lower temperature (i.e. 20°C, 17°C, 14°C or 10°C) resulted in a lag before growth resumed at a rate typical of the new temperature. Lag times appeared to increase with respect to the magnitude of the shift, while generation time responded to the post-shift in temperature as expected. Of particular interest, the RLT response of *E. coli* to cold shock displayed little variation across the tested temperature range (10°C: 0.42, 14°C: 0.59, 17°C: 0.44, 20°C: 0.23), indicating that *E. coli* undertakes a similar amount of “work” to adapt to each of these temperatures. Assessment of the global transcriptomic and proteomic response of *E. coli* O157∶H7 Sakai to an abrupt temperature downshift from 35°C to 14°C revealed a general decrease in the global expression of genes and proteins involved in DNA replication, transcription, translation and protein synthesis. This response parallels the observed decrease in growth rate of *E. coli* upon temperature downshift, and presumably also the necessity for saving and reallocating energy for repairing damage to the cell. Numerous changes occurred in the expression pattern of genes and proteins involved in transport and binding functions to contend with the effects of cold temperature on the bacterial cell envelope. The selective induction of specific amino acid biosynthetic pathways involved in histidine and the aromatic amino acid biosynthetic families also suggests a role for these amino acids in adaptation to; and growth under, chill temperature.

The expression pattern of the global stress response regulons provided insight into their roles, with sustained expression of the RpoS-dependent general stress response pathway and an initial transient induction in the RpoE-dependent extracytoplasmic stress response pathway at 30 min, when cultures were in the lag phase, and adapting to the conditions of growth. The results of this study highlight those global alterations in genome expression that may enable adaptation to, and growth under, conditions of chill temperature stress and will inform the development of more targeted approaches for the food industry to combat this pathogen.

## Supporting Information

File S1 **Table S1, Comparison of differentially expressed genes and proteins in *Escherichia coli* O157∶H7 Sakai during exposure to temperature shift.** Table S2, T-profiler analysis of transcriptomic and proteomic data based on JCVI CMR functional categories. Table S3, T-profiler analysis of transcriptomic and proteomic data based on Ecocyc and KEGG functional categories. Table S4, T-profiler analysis of transcriptomic and proteomic data based on major regulons. Figure S1, Venn diagram displaying a comparison of differentially expressed genes and proteins in *Escherichia coli* O157∶H7 Sakai during exposure to temperature shift.
(XLSX)
